# Identification of plaque ruptures using a novel discriminative model comprising biomarkers in patients with acute coronary syndrome

**DOI:** 10.1038/s41598-020-77413-3

**Published:** 2020-11-19

**Authors:** Hyungdon Kook, Duck Hyun Jang, Jong-Ho Kim, Jae-Young Cho, Hyung Joon Joo, Sang-A Cho, Jae Hyoung Park, Soon Jun Hong, Cheol Woong Yu, Do-Sun Lim

**Affiliations:** 1 Division of Cardiology, Department of Internal Medicine, Korea University Anam Hospital, Korea University School of Medicine, #73, Inchon-ro, Seongbuk-gu, Seoul, 02841 Korea; 2 Department of Cardiovascular Medicine, Regional Cardiocerebrovascular Center, Wonkwang University Medical Center, Iksan, Korea; 3 Health Insurance Review and Assessment Service, Wonju, Korea

**Keywords:** Cardiovascular biology, Biomarkers, Cardiology

## Abstract

Soluble lectin-like oxidized low-density lipoprotein receptor-1 (sLOX-1), neutrophil gelatinase-associated lipocalin (NGAL), and matrix metalloproteinase-9 (MMP-9) are inflammatory biomarkers involved in plaque destabilization resulting in acute coronary syndrome (ACS). This study aimed to investigate the diagnostic value of a combination of biomarkers to discriminate plaque ruptures in the setting of ACS. Eighty-five ACS patients with optical coherence tomography (OCT) images of the culprit plaque were included and categorized into two groups: ACS with plaque rupture (Rupture group, n = 42) or without plaque rupture (Non-rupture group, n = 43) verified by OCT. A discriminative model of plaque rupture using several biomarkers was developed and validated. The Rupture group had higher white blood cell (WBC) counts and peak creatine kinase-myocardial band (CK-MB) levels (13.39 vs. 2.69 ng/mL, p = 0.0016). sLOX-1 (227.9 vs. 51.7 pg/mL, p < 0.0001) and MMP-9 (13.4 vs. 6.45 ng/mL, p = 0.0313) levels were significantly higher in the Rupture group, whereas NGAL showed a trend without statistical significance (59.03 vs. 53.80 ng/mL, p = 0.093). Receiver operating characteristic curves to differentiate Rupture group from Non-rupture group calculated the area under the curve for sLOX-1 (p < 0.001), MMP-9 (p = 0.0274), and NGAL (p = 0.0874) as 0.763, 0.645, and 0.609, respectively. A new combinatorial discriminative model including sLOX-1, MMP-9, WBC count, and the peak CK-MB level showed an area under the curve of 0.8431 (p < 0.001). With a cut-off point of 0.614, the sensitivity and specificity of plaque rupture were 62.2% and 97.6%, respectively. The new discriminative model using sLOX-1, MMP-9, WBC count, and peak CK-MB levels could better identify plaque rupture than each individual biomarker in ACS patients.

## Introduction

Acute coronary syndrome (ACS) is a major concern for morbidity and mortality in patients suffered by ischemic heart disease^1, 2^. A complex and diverse process, including endothelial dysfunction, vascular inflammation, and hypercoagulability, result in atherosclerosis and plaque destabilization leading to ACS^3, 4^. These pathophysiological components of coronary artery disease (CAD) may be detected by several biomarkers. Consequently, biomarker levels may be associated with the severity of CAD and hence may forecast the occurrence of adverse cardiovascular events or differentiate severe events from less severe ones in patients with CAD. Such associations have already been investigated for several biomarkers, such as C-reactive protein (CRP)^5–7^, soluble lectin-like oxidized low-density lipoprotein (LDL) receptor-1 (sLOX-1), matrix metalloproteinase-9 (MMP-9), and neutrophil gelatinase-associated lipocalin (NGAL).

sLOX-1 is implied to be linked with vulnerable plaque and subsequent plaque rupture, and a previous study has reported that it may discriminate between ACS with and without plaque rupture^8^. Other studies have shown that MMP-9 indicates atherosclerotic plaque rupture or vulnerability by elastin degradation, which advances breakdown of the thin fibrous caps of plaques^9, 10^. Thus, MMP-9 activity is thought to be the key to ACS development. On the other hand, NGAL may be associated in the progression of atherosclerosis via endothelial dysfunction, inflammatory processes, and matrix degradation, leading to atherosclerotic plaque instability by modulating the activity of MMP-9^11–13^. In the presence of CAD, plasma NGAL level is significantly elevated and correlates with its severity and is notably higher in patients with acute myocardial infarction than in those with stable CAD^13, 14^.

While the most common cause of intra-coronary thrombosis is plaque rupture, which found in approximately 50% of patients with ACS^15–17^, other causes include plaque erosion, calcified plaque, tight stenosis, intramural hematoma, and spontaneous dissection. Recent studies revealed that ACS patients presenting with plaque rupture have worse prognosis^18, 19^. Therefore, differentiation between ruptured versus non-ruptured plaques in the culprit lesions is crucial for risk stratification and treatment strategy for patients with ACS.

Optical coherence tomography (OCT) has high resolution power and can discriminate several plaque characteristics of culprit lesions that result in ACS^20–25^. However, it is difficult to achieve timely risk stratification because OCT requires invasive and time-consuming coronary angiography.

Therefore, the identification of plaque ruptures without invasive testing in patients with ACS is necessary for quick risk assessment and appropriate management selection. Until now, data on the relationship between plaque rupture and serum biomarker levels are scarce.

In the present study, we sought to derive and validate a new discriminative model using several biomarkers for detecting plaque rupture in patients with ACS verified by OCT.

## Methods

### Study population and definitions

We screened 120 consecutive patients with ACS who arrived at the emergency room (ER) within 24 h after the onset of symptoms and underwent urgent percutaneous coronary intervention (PCI) between December 2014 and December 2015 at Korea University Anam Hospital. After the culprit lesions of ACS were identified based on coronary angiography findings, OCT examination of the culprit plaques was performed before PCI. Patients who did not undergo urgent PCI or those with cardiogenic shock, pulmonary congestion, fatal arrhythmia, or renal failure on regular hemodialysis were excluded from the study, as were those with inadequate OCT images due to massive residual thrombi or who required pre-dilation before OCT imaging^8^. As a result, 85 patients with appropriate OCT images were finally analyzed and categorized into two groups, namely, ACS with plaque rupture (Rupture group, n = 42) and ACS without plaque rupture (Non-rupture group, n = 43), verified by OCT^8^. The study protocol was approved by the Korea University Hospital Institute Review Board (2019AN0170), and written informed consent was obtained from all participants or their legal representatives. The study also complied with the Declaration of Helsinki.

ACS was defined as prolonged typical angina at rest (≥ 20 min) with significant coronary artery lesions confirmed on coronary angiography^8^. ST-segment elevation myocardial infarction (STEMI) was defined as ACS with new (or presumably new) ST-segment elevation (≥ 0.1 mV) in two or more contiguous leads on electrocardiography and elevated cardiac troponin^26^. Non-STEMI (NSTEMI) was defined as ACS with new (or presumably new) ST-segment deviation (≥ 0.05 mV) or T-wave inversion (≥ 0.2 mV) in two or more contiguous leads on electrocardiography and elevated cardiac troponin^8^.

### Blood sampling and measurement of biomarkers

Peripheral blood samples were collected from patients with ACS just before the PCI through the femoral or radial artery into a BD SST II Advance Tube (Becton, Dickinson and Company, Franklin Lakes, NJ, USA). Blood samples were allowed to clot for 30 min before 15 min of centrifugation at 1500×*g*. The serum was removed, aliquoted, and stored at − 80 °C prior to the determination of serum sLOX-1, NGAL, and MMP-9 levels using enzyme-linked immunosorbent assay (ELISA). Serum sLOX-1 levels were assessed using the Human LOX-1 ELISA Kit (Cell Biolabs, Inc., San Diego, CA, USA). The lower limit of detection for sLOX-1 was 40 pg/mL^27^. Total human MMP-9 level was determined using the Quantikine Human MMP-9 Immunoassay (R&D Systems, Inc., Minneapolis, MN, USA). The minimum detectable dose of MMP-9 was 0.156 ng/mL. Intra- and inter-assay coefficients of variation for the MMP-9 immunoassay were 1.9–2.9% and 6.9–7.9%, respectively. Serum NGAL levels were determined using the Quantikine Human Lipocalin-2/NGAL Immunoassay (R&D Systems, Inc.). The minimum detectable dose of NGAL was 0.012 ng/mL. The intra- and inter-assay coefficients of variation for the NGAL immunoassay were 3.1–4.4% and 5.6–7.9%, respectively. Serum high-sensitivity CRP levels were measured using latex-enhanced nephelometry (N-Latex CRP II; Siemens Healthcare Diagnostics, Tokyo, Japan). Serum high-sensitivity troponin I levels were measured using an electrochemiluminescence immunoassay (ECLusys Troponin hs; Roche Diagnostics, Tokyo, Japan). The assays were performed according to the manufacturer’s instructions. Each serum sample was evaluated twice. ELISA-based measurements were obtained using the Versa Max Microplate Reader (Molecular Devices Corporation, Sunnyvale, California, USA) at an optical density of 450 nm. Peripheral blood samples to measure serum creatine kinase-myocardial band (CK-MB) levels were serially obtained every 6 h after ER presentation until the CK-MB value began to decline, using an anti-human CK-MB monoclonal antibody (N-assay L CK-MB Nittobo; Nittobo Medical Co., Ltd., Fukushima, Japan).

### OCT image acquisition and analysis

After aspiration of the thrombi using a thrombectomy catheter, whenever possible, the culprit lesion was examined using an OCT imaging catheter. OCT imaging of the culprit lesions (30 mm in length) was acquired with a frequency-domain OCT C7XR system and the Dragon Fly catheter (Lightlab Imaging/St. Jude Medical)^8^. In the C7XR system, a 2.7-F OCT imaging catheter was carefully advanced distal to the culprit lesion. The automated pullback was performed at 20 mm/s, while blood was displaced by a short injection of contrast media through the guiding catheter. The images were digitally stored for offline analysis. All OCT images were analyzed in the Korea University Anam Hospital core laboratory by two cardiologists (Jae-Young Cho and Hyung Joon Joo) who were blinded to the angiographic data and clinical presentations. When there was discordance between the observers, a consensus was obtained from an independent cardiologist.

### Definition of plaque morphology by OCT

Plaque rupture was defined as the presence of a fibrous cap discontinuity and cavity formation in the plaque^28^. Ruptured plaques usually have an extensive lipid core and a thin fibrous cap. The fibrous cap is the thinnest part at the rupture site, and the plaque cavity indicates the loss of lipid core due to rupture. Plaque erosion was defined as the presence of an irregular luminal surface without plaque rupture in OCT images^28^. A calcified nodule was defined as the protrusion of a signal-poor or heterogeneous region with a sharply delineated border. A thrombus was defined as a protrusion inside the lumen of the artery with signal attenuation. A white thrombus, which consists mainly of platelets, was identified as signal-rich, low-backscattering protrusions in the OCT image, while red thrombus was identified as high-backscattering protrusions inside the lumen of the artery with signal-free shadowing in the OCT image. Lipid cores were defined as diffusely bordered, signal-poor regions.

### Quantitative coronary angiography

Coronary angiography was performed by engaging a Judkins catheter after puncturing the radial artery or femoral artery. Coronary angiograms were analyzed using the Cardiovascular Angiography Analysis System (Pie Medical Imaging B.V., Maastricht, the Netherlands). The reference diameter, minimum lumen diameter, diameter stenosis, area stenosis, and lesion length were measured with a computerized quantitative analyzer using a caliper.

### Statistical analysis

Continuous variables are presented as mean ± standard deviation and were compared using Student’s t-test or Wilcoxon test and categorical variables using the chi-square test. Because sLOX-1, high-sensitivity CRP, troponin I, and CK-MB levels were not normally distributed, these values are presented as median and interquartile range (IQR) and were compared using the Wilcoxon test. Correlations between each biomarker level and the peak CK-MB level were analyzed using Spearman’s rank order correlation test. The receiver operating characteristic (ROC) curve was used to assess whether the biomarkers (sLOX-1, NGAL, MMP-9) and white blood cell (WBC) count could differentiate between ruptured and non-ruptured plaques in patients with ACS. An area under the curve (AUC) of 1.0 indicated a test of perfect diagnostic value, whereas an AUC of 0.5 indicated no diagnostic value. The new discriminative model to detect plaque ruptures, which was built on multivariate logistic regression, was a score system ranging from 0 to 1 using a combination of sLOX-1, CK-MB, and MMP-9 levels and WBC count. The model equation formula is described below.

$$\widehat{y}=\frac{\begin{array}{c}exp(-3.0137 + 0.00632\times LOX-1 +0.1843\times WBC+0.00844\times CKMB -0.0086\times MMP-9 )\\ \end{array}}{1 + \mathrm{exp}(-3.0137 + 0.00632\times {\varvec{L}}{\varvec{O}}{\varvec{X}}-1 +0.1843\times {\varvec{W}}{\varvec{B}}{\varvec{C}}+0.00844\times {\varvec{C}}{\varvec{K}}{\varvec{M}}{\varvec{B}} -0.0086\times {\varvec{M}}{\varvec{M}}{\varvec{P}}-9 )}$$

The best cut-off point was calculated using the Youden’s index. When the model ROC curve was statistically significant, the curve was compared with the WBC count and sLox-1, NAGL, and MMP-9 levels. A two-sided p-value of < 0.05 was considered statistically significant. Statistical analysis was performed using SAS 9.3 (SAS Institute Inc., Cary, NC, USA).

## Results

### OCT findings in patients with ACS

**Figure 1:**
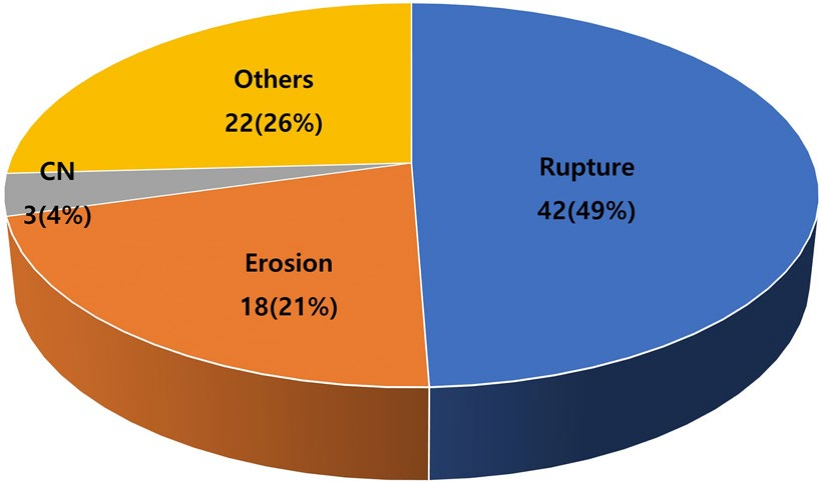
Composition of plaque morphology findings confirmed by OCT (n = 85). *CN* calcified nodule.

Plaque morphology findings confirmed by OCT in all 85 patients with ACS are shown in Fig. 1. Plaque rupture (49.4%) was the most prevalent finding, followed by plaque erosion (21.2%). The other findings included tight stenosis (22.4%), calcified nodules (3.5%), spontaneous dissection (2.4%), and intramural hematoma (1.2%).

### Baseline characteristics

Comparisons of baseline characteristics between the Rupture group and Non-rupture group are summarized in Table 1. Patients in the Rupture group were more likely to be men (88.1 vs. 69.8%, p = 0.0387) and have a clinical diagnosis of myocardial infarction (45.2 vs. 23.3%, p = 0.033) than those in the Non-rupture group. Ages were similar between the groups. Among the risk factors, body mass index (24.0, [22.9–26.7] vs. 23.4, [21.6–26.1] kg/m^2^, p = 0.0787) and incidence of diabetes mellitus (28.6 vs. 14.0%, p = 0.0991) showed a trend to be higher in the Rupture group but not hypertension, dyslipidemia, and smoking history. WBC count (8.37, [7.04–11.46] vs. 6.82, [5.79–8.8] × 10^3^/μL, p = 0.0098) was higher in the Rupture group, whereas lipid profiles showed no statistical differences between the groups.

**Table 1:** Baseline characteristics.

	Rupture group (n = 42)	Non-rupture group (n = 43)	p-value
Age, years	60.2 ± 11.5	62.7 ± 11.6	0.3253
Male sex	37 (88.1)	30 (69.8)	0.0387
BMI, kg/m^2^	24.0 (22.9–26.7)	23.4 (21.6–26.1)	0.0787
**Clinical diagnosis**			0.0825
Unstable angina	23 (54.8)	33 (76.7)	
NSTEMI	7 (16.7)	5 (11.6)	
STEMI	12 (28.6)	5 (11.6)	
Diabetes mellitus	12 (28.6)	6 (14.0)	0.0991
Hypertension	25 (59.6)	22 (51.2)	0.4382
Dyslipidemia	41 (97.6)	43 (100.0)	0.3088
**Smoking**			0.1135
Smoker	23 (54.8)	14 (32.6)	
Ex-smoker	1 (2.4)	1 (2.3)	
Never smoker	18 (42.9)	28 (65.1)	
Previous PCI	8 (19.1)	7 (16.3)	0.7378
Previous CVA	1 (2.4)	3 (4.0)	0.3171
Creatinine, mg/dL	1.05 (0.82–1.2)	0.95 (0.83–1.06)	0.1112
Pro BNP, pg/mL	158.5 (44.1–709.6)	81.3 (19.5–610.3)	0.4824
WBC, × 10^3^/μL	8.37 (7.04–11.46)	6.82 (5.79–8.8)	0.0098
Ejection fraction, %	60 (50–60)	60 (57–60)	0.1222
Total cholesterol, mg/dL	172 (59–213)	168 (130.5–201)	0.8109
LDL-cholesterol, mg/dL	113 (101–159)	111.5 (96–151)	0.5698
HDL-cholesterol, mg/dL	44.5 (35.5–83)	49 (41–62)	0.4223
Triglyceride, mg/dL	116 (89–169)	97 (66–138)	0.1403

Data are presented as mean ± SD or median (interquartile range) or n (percentage).

*BMI* body mass index, *NSTEMI* non-ST-segment elevation myocardial infarction, *STEMI* ST-segment elevation myocardial infarction, *PCI* percutaneous coronary intervention, *CVA* cerebrovascular accidents, *BNP* B-type natriuretic peptide, *WBC* white blood cell, *LDL* low-density lipoprotein, *HDL* high-density lipoprotein.

### Comparison of biomarker levels between Rupture group and Non-rupture group

**Figure 2:**
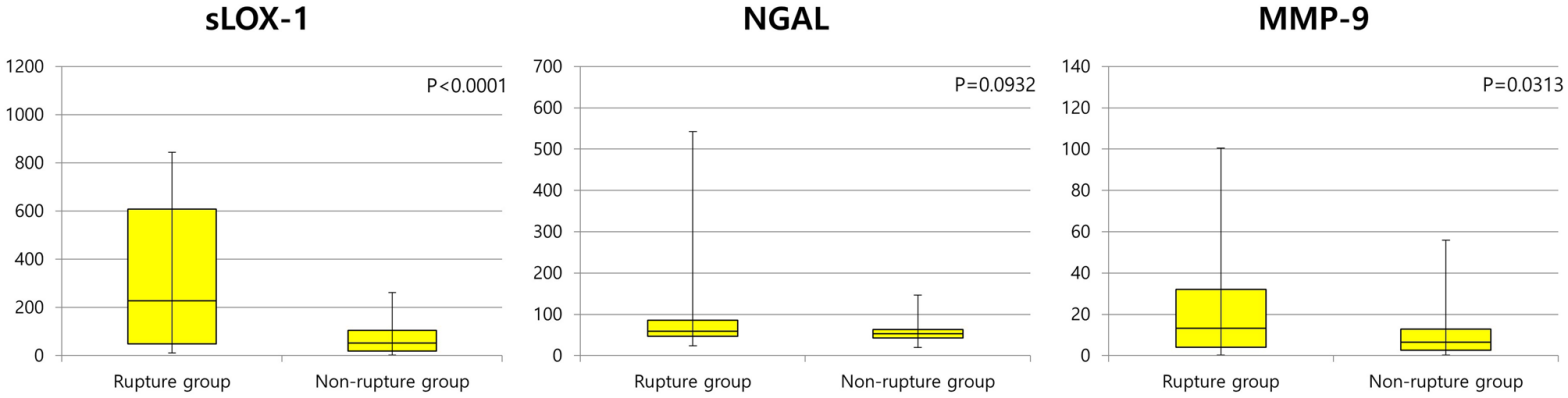
Comparison of sLOX-1, NGAL, and MMP-9 levels between the Rupture group and Non-rupture group. *sLOX-1* soluble lectin-like oxidized low-density lipoprotein receptor-1, *NGAL* neutrophil gelatinase-associated lipocalin, *MMP-9* matrix metalloproteinase-9.

Different levels of various biomarkers within each group are shown in Table 2. Median values of the peak CK-MB (13.39 [2.23–182.8] vs. 2.69 [1.65–5.19] ng/mL, p = 0.0016), sLOX-1 (227.87 [49.45–607.3] vs. 51.7 [19.6–104.3] pg/mL, p < 0.0001), and MMP-9 (13.36 [4.12–32.06] vs. 6.45 [2.56–12.94] ng/mL, p = 0.0313) levels were significantly higher in the Rupture group (Fig. 2). Scattered plot graph of peak CK-MB between two groups are presented in Supplementary Fig. S1. NGAL level (59.0 [47.2–85.9] vs. 53.1 [43.0–63.7] ng/mL, p = 0.0932) was numerically higher in the Rupture group without statistical significance. Troponin I and high-sensitivity CRP levels were not different between the groups. This trend was maintained even when comparing Non-rupture group and Rupture group only in MI patients. Comparing biomarker levels between Non-rupture group and Rupture group in patients with MI are presented in Supplementary Table S1.

**Table 2:** Comparison of biomarker levels between Non-rupture group and Rupture group in patients with ACS.

	Rupture group (n = 42)	Non-rupture group (n = 43)	p-value
Troponin I (ng/mL)	0.21 (0.12–5.82)	0.19 (0.1–0.92)	0.7244
Peak CK-MB (ng/mL)	13.39 (2.23–182.8)	2.69 (1.65–5.19)	0.0016
High-sensitivity CRP (mg/L)	1.35 (0.62–3.94)	1.08 (0.63–4.13)	0.6635
sLOX-1 (pg/mL)	227.87 (49.45–607.3)	51.7 (19.6–104.3)	< .0001
NGAL (ng/mL)	59.0 (47.2–85.9)	53.1 (43.0–63.7)	0.0932
MMP-9 (ng/mL)	13.36 (4.12–32.06)	6.45 (2.56–12.94)	0.0313

Data are presented as median (interquartile range).

*ACS* acute coronary syndrome, *CK-MB* creatine kinase-muscle/brain, *CRP* C-reactive protein, *sLOX-1* soluble lectin-like oxidized low-density lipoprotein receptor-1, *NGAL* neutrophil gelatinase-associated lipocalin, *MMP-9* matrix metalloproteinase-9.

### Correlation between peak CK-MB and biomarker levels

**Figure 3:**
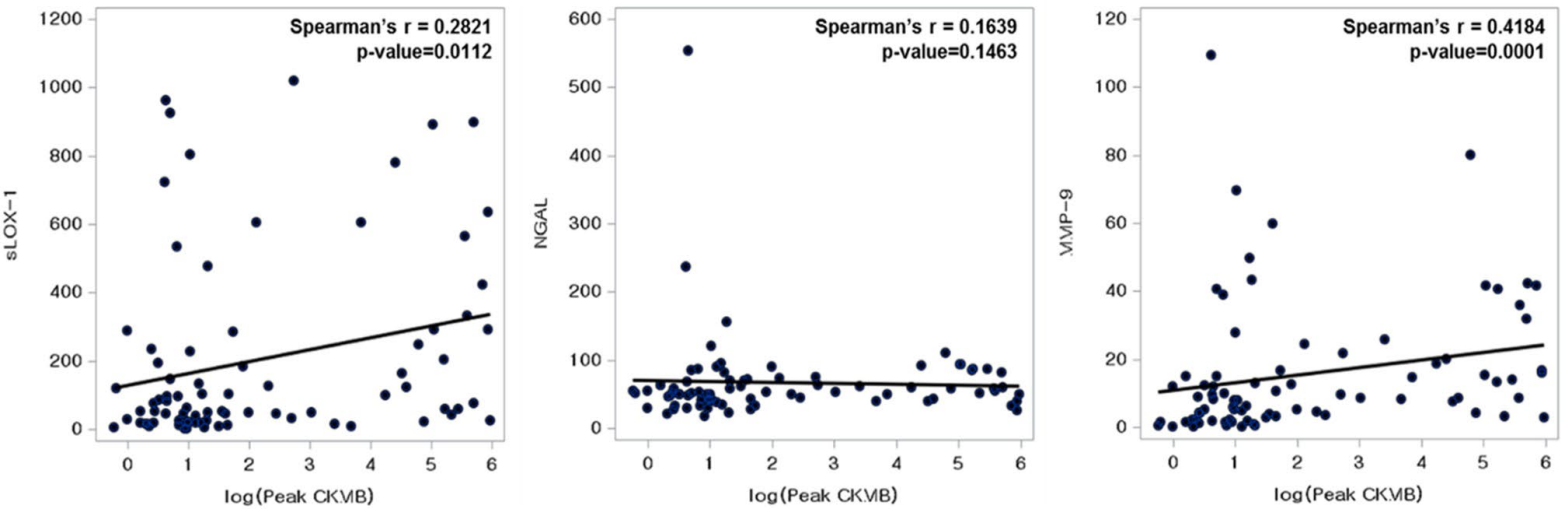
Correlation between the peak CK-MB level and biomarker levels. *CK-MB* creatine kinase-muscle/brain, *sLOX-1* soluble lectin-like oxidized low-density lipoprotein receptor-1, *NGAL* neutrophil gelatinase-associated lipocalin, *MMP-9* matrix metalloproteinase-9.

A correlation between the peak CK-MB and biomarker levels was observed (Fig. 3). sLOX-1 (Spearman ρ = 0.2821, p = 0.011) and MMP-9 (Spearman ρ = 0.418, p = 0.0001) levels, but not NGAL (Spearman ρ = 0.164, p = 0.146) level, were significantly and proportionally correlated with the peak CK-MB level.

### A new discriminative model using sLOX-1, MMP-9, and peak CK-MB and WBC to differentiate Rupture group from Non-rupture group

**Figure 4:**
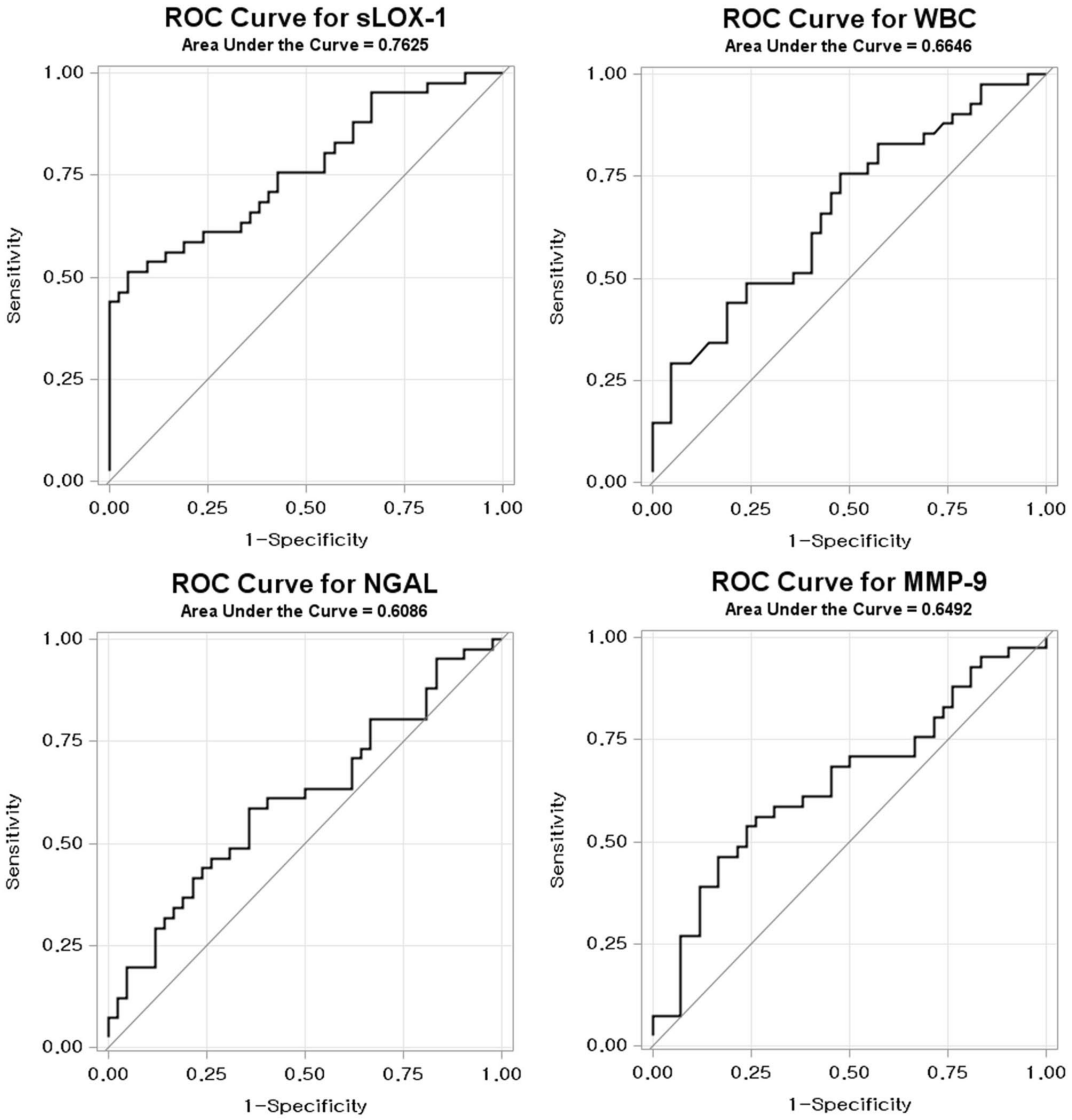
Receiver operating characteristic (ROC) curves for differentiating Rupture group from Non-rupture group. *sLOX-1* soluble lectin-like oxidized low-density lipoprotein receptor-1, *WBC* white blood cell; *NGAL* neutrophil gelatinase-associated lipocalin, *MMP-9* matrix metalloproteinase-9.

**Figure 5:**
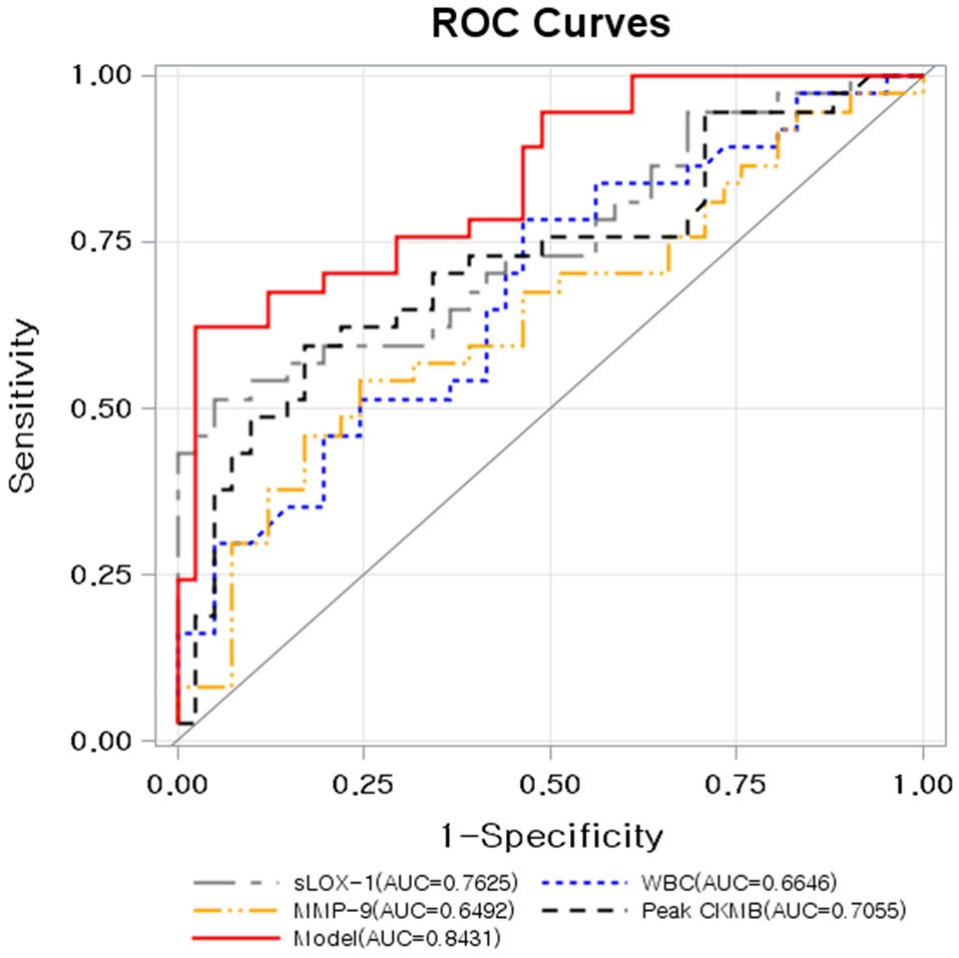
Receiver operating characteristics (ROC) curves displaying the additional discriminatory ability of the new predictive model using sLOX-1, MMP-9, and peak CK-MB levels and WBC count in combination for differentiating Rupture group from Non-rupture group. *AUC* area under the curve, *sLOX-1* soluble lectin-like oxidized low-density lipoprotein receptor-1, *MMP-9* matrix metalloproteinase-9, *CK-MB* creatine kinase-muscle/brain; and *WBC* white blood cell.

The ROC curve analysis for sLOX-1, NGAL, MMP-9, and WBC to detect Rupture group using Non-rupture group as a negative reference is shown in Fig. 4. The AUCs were 0.763 (p < 0.0001), 0.645 (p = 0.0274), 0.609 (p = 0.0874), 0.665 (p = 0.0016), and 0.701 (p = 0.0007) for sLOX-1, MMP-9, NGAL, WBC, and the peak CK-MB level, respectively. The optimal cut-off values were 236.4 pg/mL (50% sensitivity and 92.9% specificity), 12.7 ng/mL (54.8% sensitivity and 74.4% specificity), 56.0 ng/mL (57.1% sensitivity and 65.1 specificity), and 6.900/µL (75.6% sensitivity and 52.4% specificity) for sLOX-1, MMP-9, NGAL, and WBC count, respectively. The AUC value of the new discriminative model using LOX-1, MMP-9, WBC count, and the peak CK-MB level in combination was 0.843 (p < 0.0001), and the optimal cut-off value was 0.614, which was calculated using the Youden’s index (62.2% sensitivity and 97.6% specificity; Fig. 5). The ROC curve of the new predictive model to detect Rupture group in patients with ACS was superior to sLOX-1 (p = 0.0346), MMP-9 (p = 0.0002), NAGL (p = 0.0004), WBC count (p = 0.0016), and peak CK-MB level alone (p = 0.0199; Table 3).

**Table 3:** Comparison between the new predictive model and each biomarker in terms of their power in differentiating Rupture group from Non-rupture group in patients with ACS.

	AUC (95% CI)	Cut-off point	Sensitivity	Specificity	p-value
Model	0.843 (0.757, 0.929)	0.61	62.2	97.6	–
sLOX-1	0.763 (0.660, 0.865)	236.4	50	95.35	0.0346
WBC	0.665 (0.548, 0.781)	6.9	75.61	52.38	0.0016
NGAL	0.609 (0.486, 0.731)	56.0	57.14	65.12	0.0004
MMP-9	0.645 (0.524, 0.766)	12.7	54.76	74.42	0.0002
Peak CK-MB	0.706 (0.587, 0.824)	7.3	57.9	83.3	0.0199

The cut-off point is calculated using the Youden’s index.

*ACS* acute coronary syndrome, *AUC* area under the curve, *sLOX-1* soluble lectin-like oxidized low-density lipoprotein receptor-1, *WBC* white blood cell, *NGAL* neutrophil gelatinase-associated lipocalin, *MMP-9* matrix metalloproteinase-9, *CK-MB* creatine kinase-muscle/brain.

p-values are the comparison results between new predictive model and each biomarker.

## Discussion

### Study findings

This study investigated the association between circulating plasma biomarkers and plaque characteristics defined by OCT in patients with ACS. Consequently, we demonstrated that plasma sLOX-1 and MMP-9 levels, but not NGAL levels, were higher in Rupture group than Non-rupture group. In addition, we evaluated a new discriminative model comprising several biomarkers, cardiac enzymes, and WBC count to differentiate Rupture group from Non-rupture group, which displayed an AUC of 0.843 (p < 0.0001). When this model was compared with each biomarker, it showed an improved discriminatory ability to differentiate Rupture group from Non-rupture group.

The major findings of this study were as follows: (1) conventional cardiac biomarkers, including troponin I and high-sensitivity CRP, cannot differentiate between ACS with and without plaque ruptures, which is in line with previous findings^8^; (2) sLOX-1, MMP-9, and the peak CK-MB levels and WBC count have a potential to differentiate Rupture group from Non-rupture group, and among them, sLOX-1 has the greatest discriminatory ability; (3) a new discriminative model, developed by combining sLOX-1, MMP-9, and peak CK-MB levels with WBC count, could better discriminate plaque ruptures than individual biomarkers; (4) higher sLOX-1 and MMP-9 levels were well correlated with a higher peak CK-MB level and WBC count, which may reflect increased myocardial damage and subsequent inflammatory reaction in Rupture group than Non-rupture group; and (5) NGAL had no discriminatory power between Rupture group and Non-rupture group, although it is believed to be associated with poor clinical outcomes in ACS^29^.

### Biomarkers and ACS

The peak CK-MB level represents the amount of myocardial damage in ACS, which is associated with worsened long-term prognosis. In the present study, sLOX-1 and MMP-9 were significantly correlated with the peak CK-MB level, whereas NGAL was not well correlated.

MMPs, a family of zinc-containing endoproteinases, degrade the extracellular matrix components^30^. Vascular smooth muscle cells, macrophages, and endothelial cells express MMPs in accordance with inflammatory stimuli and oxidative stress. MMP-9 advances breakdown of the thin fibrous caps of plaques by elastic degradation, which indicates atherosclerotic plaque rupture or vulnerability^9, 10^. A previous study has shown that patients with polymorphisms of the MMP-9 promotor have elevated MMP-9 expression, which was associated with acute myocardial infarction^31^. Many other studies have revealed that patients with ACS have significantly higher MMP-9 levels, with the highest in patients with STEMI, than in those with stable angina or healthy controls^9, 32–35^.

However, Nishiguchi et al. reported that systemic MMP-9 level in patients with plaque rupture was equivalent to the level without plaque rupture^36^. In their study, local MMP-9 was higher in the plaque rupture group. Since the concentration of systemic MMP-9 was much dilute and lower than that of local MMP-9, more samples might have been needed to show statistical differences at lower concentrations. Also, there was a difference in the time taken for sampling between the present study and one by Nishiguchi et al., which may have influenced the results.

Several other studies have reported robust associations between MMP-9 level and the subsequent risk of cardiovascular complications, such as cardiovascular mortality and nonfatal myocardial infarction. Therefore, MMP-9 may act as not only a causative agent on plaque destabilization but also a circulating marker reflecting a proinflammatory state^37, 38^.

Plasma oxidized LDL cholesterol levels are related to the thrombogenicity of coronary lesions in patients with unstable angina^39^. LOX-1, a scavenger receptor for the uptake of atherogenic oxidized LDL in the arterial wall, is plentifully expressed in advanced atherosclerotic plaque and plays an important role in the development of oxidative stress and inflammation^40, 41^. When LOX-1 is cleaved by proteases at its proximal membrane extracellular domain following acute myocardial ischemia, sLOX-1 is released into the circulation^42, 43^. sLOX-1 may arise from activated platelets, atheroma, or endothelial cells^40^. Therefore, circulating sLOX-1 levels are increased in patients with ACS. As a result, plasma sLOX-1 levels are a useful biomarker of ACS^44^.

Taken together, MMP-9 is a causal proteinase that may contribute to plaque rupture by eroding or weakening a plaque cap and sLOX-1 is a consequent product of plaque rupture; thus, the two biomarkers directly reflect plaque rupture, and of the two, sLOX-1 has a stronger correlation with plaque rupture than MMP-9 in our study.

NGAL, a 25 kDa glycoprotein isolated from the granule of mature neutrophils, covalently binds to MMP-9 and increases MMP-9 activity^45, 46^. NGAL is expressed in smooth muscle cells, macrophages, and endothelial cells in atherosclerotic plaques^11, 47^. Several studies have shown increased NGAL levels in CAD, which imply the involvement of NGAL in the atherosclerotic process^14, 48^. In addition, a study has shown that NGAL levels were positively correlated with lesion complexity and the severity of CAD in patients with ACS^49^. However, NGAL binding to MMP-9 increases the rate of MMP-9/tissue inhibitor of metalloproteinase^46^. This stromal factor, the tissue inhibitor of metalloproteinase, inhibits the activity of MMP-9. Thus, NGAL activation plays as a modulator and leads to increased MMP-9 activity and plaque instability rather than plaque rupture itself. One study reported that NGAL is associated with long-term prognosis in patients with acute myocardial infarction because it has no direct relevance with plaque rupture but with plaque destabilization^29^.

### Clinical implications of the identification of plaque rupture in patients with ACS

Several studies have proved that plaque rupture is the main cause of ACS and worsens the prognosis compared to Non-rupture group^19, 50, 51^. Plaque rupture is indeed the most common cause of coronary thrombosis, found in nearly 50% of patients with ACS, while plaque erosion is found in up to one-third of patients with ACS^15–17^. The other underlying mechanisms of ACS include calcified plaque, tight stenosis, intramural hematoma, and spontaneous dissection. Plaque rupture is often associated with positive remodeling, large plaque burden, and red thrombus^52^. Large plaque burdens in patients with plaque rupture may lead to residual plaque after PCI, which is a known predictor of stent failure^19^. Also, a previous study reported that patients with plaque rupture may have diffuse vulnerable features throughout the whole coronary tree^18^. These mechanisms together may reflect the worsened prognoses in patients with plaque rupture. On the other hand, plaque erosion is associated with negative remodeling, modest plaque burden, white thrombus, uncommon features of a fibroatheroma, and proximal distribution^52^. Given these findings, it may be reasonable to treat patients with plaque erosion by effective antithrombotic treatment without early invasive stent implantation^53^.

However, no single biomarker can successfully differentiate plaque rupture from non-plaque rupture in patients with ACS. Intravascular imaging tools such as OCT have advantages in detecting plaque rupture in the catheterization laboratory setting but have limitations in their usage due to the requirement of invasive procedures, such as coronary angiogram, low accessibility, high cost, possible complication risks, and time consumption. Therefore, discriminating ruptured and non-ruptured plaques using novel models comprising biomarkers without invasive procedures would be valuable for rapid risk stratification and predicting the prognoses of patients with ACS. At present, since new biomarkers including sLOX-1, MMP-9 are not established as routine laboratory tests, it may take considerable time to calculate this novel discriminative model and apply it to the treatment strategy of ACS patients. In our study, as described in the Methods section, it took about an hour to obtain each biomarker value after blood sampling. Calculating model and decision-making process would be faster if biomarker values are obtained though vein sampling as soon as patients with suspected ACS arrives at the emergency room. However, assumption of that values of biomarkers are similar between arterial sampling and venous sampling should be confirmed in future studies. With future technical advances to make inflammatory marker testing easier, more accurate and faster than it is today, we expect that the model developed in this study can be useful for identifying plaque ruptures quickly in bed-side setting and establishing treatment strategy accordingly. By quickly suspecting plaque rupture in an ACS setting using this discriminative model, the decision-making process for early invasive treatment strategies might be initiated earlier and more aggressive approach to use intravascular imaging tool to get stent optimization would be recommended, thereby improving the likelihood of better clinical outcomes. We believe that the present study is meaningful as it shows the possibility to create a more powerful model that can identify plaque rupture in patients with ACS using the existing biomarkers and laboratory data without invasive imaging tools.

### Limitations

This study has several limitations. First, this study has all inherent limitations of a small-sized observational study. Second, we excluded patients requiring pre-dilation before OCT imaging. Third, because OCT signals may be interfered with by residual microthrombi, patients in the Non-rupture group may have had minor plaque rupture that was undetectable by OCT. Fourth, risk factors including sex, diabetes mellitus, and body mass index, which were different in both groups, may have affected the results of biomarkers. Fifth, although most sampling was performed just before the PCI procedure, the exact timing of blood sampling was not specified. Therefore, the effect of ischemic time on blood concentrations of biomarker could not be corrected because the time from symptom onset to sampling could not be accurately determined. However, because this study was conducted at a single institution with similar practices in treating patients with ACS and there was no difference in the ischemic time between the groups, the time from ischemic onset to sampling was likely to be similar in both groups. Finally, because blood sampling was performed through the femoral or radial artery rather than the coronary artery, blood concentrations of these biomarkers may have been affected by vascular beds other than the coronary artery. Therefore, sampling through the coronary artery is necessary to avoid this confounding factor in future research.

In conclusion, the new discriminative model using sLOX-1, MMP-9, and peak CK-MB levels and WBC count may identify plaque ruptures in an ACS setting. Further larger-sized prospective studies to externally validate the findings of this study are warranted.

## Supplementary information

Supplementary Information.
