# Supplementary material for: Identification of plaque ruptures using a novel discriminative model comprising biomarkers in patients with acute coronary syndrome
Source: Sci Rep. 2020 Nov 19;10:20228. doi: 10.1038/s41598-020-77413-3 (PMC7677551; doi:10.1038/s41598-020-77413-3)
Supplement: Supplementary file 1 — Supplementary Information. [file 41598_2020_77413_MOESM1_ESM.pdf]

Identification of plaque ruptures using a novel discriminative model comprising biomarkers in patients with acute coronary syndrome

Hyungdon Kook[1], MD, Duck Hyun Jang[1], MD, Jong-Ho Kim, PhD[1], Jae-Young Cho[2], MD, Hyung Joon Joo[1], MD, Sang-A Cho[3], MS, Jae Hyoung Park[1], MD, Soon Jun Hong[1], MD, *Cheol Woong Yu[1], MD, *Do-Sun Lim[1], MD

[1]Division of Cardiology, Department of Internal Medicine, Korea University Anam Hospital, Korea University School of Medicine, Seoul, Korea

[2]Department of Cardiovascular Medicine, Regional Cardiocerebrovascular Center, Wonkwang University Medical Center, Iksan, Korea

[3]Health Insurance Review and Assessment Service, Wonju, Korea

*Last two authors are co-corresponding authors.

Supplementary Figure 1. Scattered plot of peak CK-MB between Rupture group and Non-rupture group. Red lines indicate median value with interquartile range.

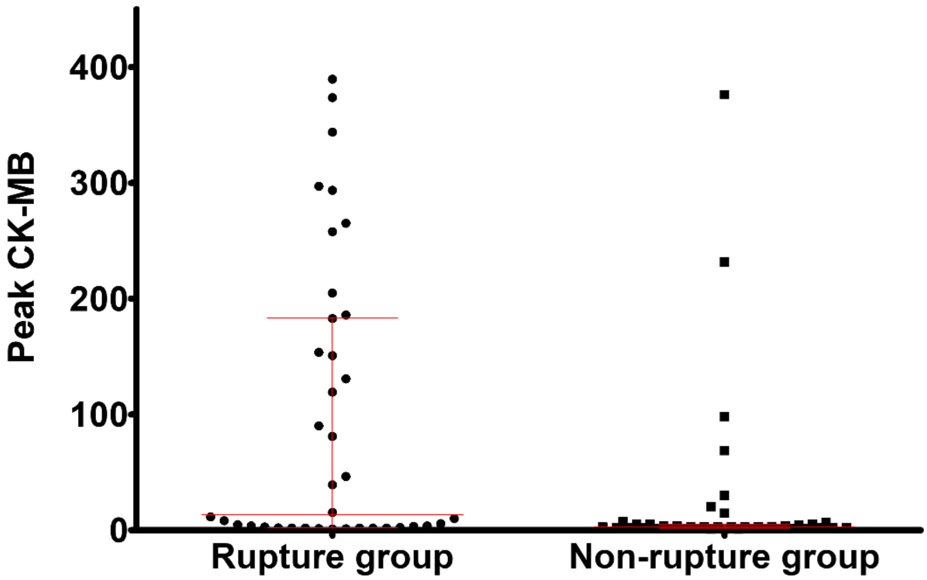

Supplementary Table 1. Comparison of biomarker levels between Non-rupture group and Rupture group in patients with MI.

	Rupture group (n=19)	Non-rupture group (n=10)	p-value
Troponin I, ng/mL	13.90±34.00	17.64±34.52	0.791
Peak CK-MB, ng/mL	179.27±123.43	84.30±124.76	0.060
High-sensitivity CRP (mg/L)	10.42±22.84	12.34±27.53	0.862
sLOX-1 (pg/mL)	405.44±324.54	74.61±84.52	<0.001
NGAL (ng/mL)	66.15±22.74	56.73±20.34	0.282
MMP-9 (ng/mL)	23.69±19.11	10.98±7.98	0.018
WBC, x10^3/μL	11.27±4.08	8.75±2.37	0.045

Data are presented as mean±standard deviation. MI = myocardial infarction; CK-MB = creatine kinase-muscle/brain; CRP = C-reactive protein; sLOX-1 = soluble lectin-like oxidized low-density lipoprotein receptor-1; NGAL = neutrophil gelatinase-associated lipocalin; MMP-9 = matrix metalloproteinase-9
